# Capital Punishment for Murder Scripturally Considered

**Published:** 1857-04-01

**Authors:** J. F. Denham


### Art. III.—CAPITAL PUNISHMENT FOR MURDER SCRIPTURALLY CONSIDERED.

BY THE REV. J. F. DENHAM, M.A., F.R.S., ETC.

We believe that there is a great and increasing repugnance to the infliction of the extreme penalty of the law, even in the case of murder, among intelligent and reflecting members of the community. The considerate observer of passing events remarks, that so far from that penalty seeming to act as a prevention of the crime, it is common to find unusually numerous instances of it occurring even during the trial or just after the execution of some homicide whose enormous or repeated guilt, and whose dreadful fate, are fully known to the entire population. The *psychologist* suspects that there is something dangerously suggestive of the act of murder (or, at least, promotive of indifference to human life) in every public execution for it; and, not only as actually witnessed, but as even read of, in the minute details of the scene furnished by the newspapers, &c.; he accordingly receives with dismay the information certified by the *Times*, October 23rd, 1856, that "there were sold of the last dying speech, confession, and behaviour of Good, 1,650,000 copies; of Courvoisier, 1,666,000; of the Mannings, 2,000,000; of Rush, 2,500,000; of Greenacre, 2,666,000; and that the trash sold with reference to Palmer's case must have greatly exceeded any of the above sales." The student in moral and political science, whom we will take to be represented by Paley,* lays down the principle that "the proper end of human punishment is not the satisfaction of justice, but the prevention of crimes—meaning

* Moral and Political Philosophy. Book VI. c. ix. Of Crimes and Punishments.

by the satisfaction of justice the retribution of so much pain for so much guilt; which is the dispensation we expect at the hand of God, and which we are accustomed to consider as the order of things that perfect justice dictates or requires. In what sense, or whether with truth in any sense, justice may be said to demand the punishment of offenders, he does not inquire; but he asserts that this *demand* is not the motive or occasion of human punishments. The fear lest the escape of the criminal should encourage him, or others by his example, to repeat the same crime, or to commit different crimes, is the sole consideration which authorises the infliction of punishment by human laws. Now, that, whatever it be, which is the cause and end of the punishment ought, undoubtedly, to regulate the measure of its severity. But this cause appears to be founded, not in the guilt of the offender, but in the necessity of preventing the repetition of the offence. The crimes must be prevented by some means or other; and, consequently, whatever means appear necessary to this end, whether they be proportionable to the guilt of the criminal or not, are adopted rightly, because they are adopted upon the principle which *alone* justifies the infliction of punishment at all. From the same consideration it also follows, that punishment ought not to be employed, much less rendered severe, when the crime can be prevented by any other means. Punishment is an evil to which the magistrate resorts only from its being necessary to the prevention of a greater. This necessity does not exist when the end may be attained, that is, when the public may be defended from the effects of the crime, by any other expedient. The right of punishment results from the necessity of preventing the crime." It follows, consequently, from the principles of moral and political philosophy, that if capital punishment for murder fails as a preventive of the crime, it ought to be abolished, and some more effectual preventive be substituted for it; and that certain popular modes of thinking and speaking are inadmissible, derived from the principle of *retaliation*, such as "the satisfaction of a natural instinct," "public indignation," against the offender and his offence, &c. *The social economist* doubts the expediency of capital punishment for murder, upon observing the conduct, language, &c., of by far the greatest proportion of persons assembled to witness the spectacle, evidently attracted thither only for the sake of gratifying certain pernicious emotions; and arguing from even his own consciousness, he doubts the possible beneficial connexion between a remote, transient, and exciting scene, and the dispositions and principles of any man about to commit murder. *The respectable provincial citizen* deplores the desecration of public decency and tranquillity occasioned in every county town by the invariable collection of the dissolute and abandoned from all quarters, around the gallows. *The London tradesman or manufacturer* is disgusted, and his affairs are frequently deranged by the irrepressible desire of his apprentices and workmen to go to "hang fair," as an "execution morning" has been called, for many years at least, among certain working classes of the metropolis. He hears with regret of boys from parochial schools and other children, and even female children, forming a portion of "the crowd," and of some of them being elevated on the shoulders of adult spectators to witness the fall of the criminal.* *Religious persons*, of various communions, lament the degradation inflicted on our common nature by the public destruction of human life by the hangman. *All right-minded persons* would confess, we presume, that could capital punishment for murder be *legitimately* abandoned, great relief would be afforded to their feelings, which, though not now, as in past years, harrowed up by reading or hearing of the bi-weekly execution of several fellow-creatures at a time for forging a twenty-shilling note, or for purloining to that amount from a dwelling-house, or for stealing a sheep, or a horse, or for some other of the 159 offences besides murder, formerly declared, by the statutes, to be capital,† are still shocked at intervals by hearing or reading of the public extinction of the convicted murderer's life. But persons belonging to the foregoing classes are generally overawed by the well-known precept delivered by God to Noah and his sons just after the deluge, "Whoso sheddeth man's blood, by man shall his blood be shed: for in the image of God made he man."‡ This precept, or rather the popular interpretation of it is, we believe, the chief obstacle to the abolition of capital punishment for murder. Accordingly it is sometimes urged by religious persons in respectable society, &c., that if capital punishment for murder were to be discontinued the word of God would be disobeyed, and the nation at large would set itself in direct and wilful opposition to a plain command of revelation. This passage is even quoted by Blackstone in his *Commentaries*, as the Scriptural command for putting the murderer to death. No one goes to the Levitical law for a command to this effect, because all persons are aware of the inconsistency of "picking and choosing" from that law, and that a full compliance with it would necessitate the introduction of some new modes of capital punishment

* The well-known appetite of the lower orders for "the horrible and awful" was last year pandered to most effectually by the *histrionic representation* of Palmer's execution, in effigy, "in the same clothes," with "the face taken from a cast," and enacted by "the same hangman from Dudley," got up by a publican in his grounds, in Staffordshire, for the amusement of his customers, twice a day, at sixpence each. —*Globe Newspaper*.

† Ruffhead's Index to the Statutes (tit. Felony), and the acts since made.

‡ Genesis ix. 6.

among us, or revive the use of others long ago discontinued. No one, we presume, wishes to see "stoning" introduced, or "burning" restored. All persons, we believe, would revolt from the infliction of death on the man who should gather sticks on the Sabbath,* or, that should curse or smite his father or his mother,† or on the adulterer or adulteress,‡ the worshipper of false gods,§ on the manstealer,|| and for numerous other offences, not now capital in this country. Nor can any other than an inferential, indirect and defective argument for capital punishment, even in the case of murder, be derived from Christianity,¶ which in its original constitution as represented in the New Testament "is not a kingdom of this world," and at its first propagation did not, for reasons derived from its circumstances as well as its constitution, interfere with the legal code of heathen nations, but wisely, if not necessarily, enjoined only on its disciples to be subject to the existing higher powers; but yet most certainly does not forbid Christian legislators and subjects in Christian countries to endeavour to assimilate the public laws to the rational and humane genius of their religion.

That one precept then, given by God to Noah and his sons, when they "went forth of the Ark," is commonly supposed to contain the entire Scriptural authority and command for the infliction of death upon the murderer by Christian nations at the present hour. "It is," said the *John Bull* newspaper last year, "the *only* and *all-sufficient answer* to the various fallacies put forth by those who would expunge capital punishment from our penal code." We presume, however, that no dispassioned person would object to consider any reasoning that might be offered with a view to ascertain the real import and proper application

* Num. xv. 32. † Lev. xx. 9.; Ex. xxi. 15.
‡ Lev. xx. 10. § Deut. xiii. 6, &c. || Ex. xxi. 16.

¶ Thus, because St. Paul enjoins on the Roman and foreign Christians living under the pagan empire, in the time of Nero, "to be subject unto the higher powers," and remarks, "if thou do that which is evil, be afraid, for he beareth not *the sword* in vain;" and because it is also *taken for granted* that "the sword" here means the power of life and death, it *is inferred* that the apostle sanctions capital punishment as a *principle.* But he partly explains his meaning by simply saying "a revenger to execute wrath upon him that doeth evil." It should also be remembered, that all crimes against the ancient Roman power were not capital. This inferential argument would, however, in all fairness lead to the conclusion that all the crimes that *were* so punished by the Roman power should be similarly punished in Christian England; and that not the sceptre but the sword should accordingly be the emblem of British monarchy. The sword, *pugio,* as worn by Roman emperors, was simply the emblem of imperatorial power *in general;* adopted, naturally, by a military nation. Nor does St. Paul represent the Roman "power" merely as an executioner, but as "the minister of God for the good of the people." No authority for capital punishment can be inferred, therefore, from this symbol, any more than from the sword carried at this day before our chief civic authorities, &c. Nor should the distinction pointed out by Paley be overlooked, that "what St. Paul designates 'the ordinance of God' is, without any real repugnancy, by St. Peter denominated '*the ordinance of man.*'"

of that precept; because there is absolutely no other mode whereby the true sense and proper use of any passage of Scripture whatever can be ascertained; nor is there any medium between the willingness to investigate the meaning and intention of any portion of Holy Writ with candour and patience, and a blind superstition that is liable to be misled by the sound of words into any possible absurdity of belief and conduct. We now then invite the careful and unbiassed attention of the reader to some observations sanctioned, as will be seen, by *eminent Biblical scholars*, upon the precept in question, and respecting the obligation it is commonly considered to impose absolutely, on Christian legislators to put the murderer to death. First. Our attention will be directed to the words of the precept, which, with its context, reads as follows:—"And surely your blood of your lives will I require; at the hand of every beast, or rather *soul*,* will I require it, and at the hand of man; at the hand of every man's brother will I require the life of man." Then comes the recapitulation of the subject previously enunciated, so frequent in the book of Genesis—"Whoso sheddeth man's blood, by man shall his blood be shed; for in the image of God made he man."†

Now there is, we think, something that must strike every attentive reader of even the English version of this passage, as remarkable in the introduction into it of the word *brother*; "at the hand of every man's *brother* will I require the life of man"—literally, and at the hand of *the* man, at the hand of a man his brother will I require the life of man. Dr. Boothroyd translates—"from every man's own brother will I demand an account of the life of man."‡ Dr. Geddes—"from a man's own brother."§ The Samaritan copy and eight manuscripts, as also the Syriac and Vulgate all read—of a man and of his brother. "The common Greek text," as Dr. Geddes observes, "is corrupted and unintelligible; nor do the manuscripts afford anything like a decent correction. The comma is admirably well rendered by the Greek of Venice προς χειρος ανδρος του αδελφου αυτου," literally—from or by the hand of a man and his brother, or of a man the brother of him. Now we cannot allow ourselves to depart from the letter of this divine enactment; and we, therefore, reject the interpretation which would generalize this word "brother" into the

* "According to tradition the first part of this text prohibits suicide, and the second half homicide. Where no adjunct is coupled with חיה, that word invariably relates to *the soul of man*. The rule holds good here. Hence then we have the satisfaction to find in the Sacred Scriptures this early and perfect indication of a punishment to the soul after death, and the necessary *sequitur*—its immortality." New Translation, by the Rev. D. A. De Sola, and the Rev. Morris J. Raphall. Vol. i. pp. 34, 52. London. 1844.

† Genesis, ix. 5, 6.

‡ Family Bible in loc.

§ Critical Remarks on the Hebrew Scriptures.

sense of "brother man," or would gloss as follows, "though the murderer be as nearly related as a brother, he shall be punished." Nor do we see any assistance given to the interpretation of this precept in the marginal references appended to it in the English version, one of which is to Acts xvii. 26—"God hath made of one blood all nations;" two, consist of predictions, that "they that take the sword shall perish with the sword" (Matt. xxvi. 52). "He that killeth with the sword must be killed with the sword" (Rev. xiii. 10); and the rest are to the incorporation of the precept in the Levitical law. We adhere strictly to the terms of the enactment, and we plead that such an adherence is essential to the legitimate and safe use of all enactments, human and divine. We are, indeed, willing to allow that the word here translated brother includes kinsmen of various degrees of consanguinity (comp. Gen. xiii. 8 אנשים אחים), clansmen (1 Chron. vi. 39, &c.); but we say that this precept delegates the infliction of death upon the murderer, either by the hand of the "brother" literally, or by some other nearest kinsman of the murdered man. In short, we believe the import of the precept to be conveyed in the literal sense of it, and that it contains the appointment of "the ancient and universal law of BLOOD REVENGE," called by the Hebrews גאל הדם goel-hadam; whereby, as Jahn observes on this precept, "the punishment of homicide devolved on the brother or other nearest relation of the person whose life had been taken away. In case he did not slay the guilty he was considered infamous. Hence the application of the Hebrew *Goel*—*i.e.*, spotted or contaminated, which he bore till the murder was revenged." He adds. "To change a law, however, or practice of long standing, is a matter of no little difficulty. Moses, therefore, left it as he found it; but he endeavoured, nevertheless, to prevent its abuses by the appointment of cities of refuge (Num. xxxv. 9—29; Deut. xix. 6; and Josh. xx. 3), to one of which all persons who had been the cause of death to another might flee and be protected until the case was investigated; and if found, according to the laws, guilty of homicide, the manslayer was delivered up to the avenger of blood, who was always supposed to be both prosecutor and executioner."*

Secondly. We now pause for a time to remind the reader of the universal prevalence of this law of Blood revenge in the earliest times, and among most eastern nations down to the present hour. The action of this law is first met with in the history of Abraham, where Esau, having been overheard purposing to kill Jacob his brother, Rebekah sent Jacob away to Haran, saying—"Why should I be deprived of you both in one

* Archäol. II. ii. 372 ff.

day," plainly intimating that the next of kin, which, in this case, would have been the eldest son of Ishmael, would have been bound to kill Esau, had he effected his purpose. This law appears again in the fiction practised upon David by the woman of Tekoah, where, however, it was overruled in her favour by the dictum of the king (2 Sam. xiv. 2, &c). Josephus relates the continuance of it among the inhabitants of Trachonitis.* Goguet thus describes its prevalence among the ancient Greeks :—"They had no public officer charged to look after murderers. The relations of the deceased alone had the right to pursue revenge. Homer shows it clearly (*Il.* 9, lin. 628, &c). We may add to the testimony of this poet that of Pausanias, who speaks in many places of this ancient usage (lib. v. c. 1, p. 376 ; lib. viii. c. 34, p. 669) ; an usage that appears to have always subsisted in Greece. (See Plat. *de Leg.* l. 9, p. 930, 931, and 933. Demosth *in Aristocrat.* p. 736. Pollux, lib. viii. c. 10, segm. 118)."† Mahomet, like Moses, did not abolish, but modify the law of Blood-revenge, by allowing of the acceptance of money for the forfeited life of the murderer, and at the worst by forbidding the infliction of any painful or cruel death.‡ It exists to this day among the Arabs, the peasantry of Egypt,§ the Persians, Abyssinians, Druses, Circassians, and Tartars.|| In Corsica and Sardinia this law is still in action, and known by the name of *vendetta traversa*, or mutual vengeance, having withstood all the efforts of the celebrated General Paoli to eradicate it. It is executed by even females.¶ "The law of *Thar*, or blood avenging," says Kitto, "existed *from the earliest ages*, and still by its action upon the fears of the wild tribes of the desert, and indeed of all the less civilized tribes of Western Asia, from the shores of the Red Sea to the Caucasian mountains, keeps in check their fiercer passions, and makes them backward to shed blood. By this law, the nearest relation of the slain party is bound to pursue the slayer, and to rest not—never to let his purpose sleep—till he has exacted life for life, and blood for blood." Now, this early and extensive prevalence of the law of blood revenge, is only explicable by the interpretation we have given of the precept to Noah and his sons, or rather by the literal rendering of that precept. The various gentile nations could only have thus pervasively derived it from some common origin of it; and no other such origin is assignable, except in the renewal of the

* Ant. iv. 7, 4.

† Origin of Laws, &c. Part II. Book I. Art. viii. Vol. ii. p. 71. Edinburgh.

‡ Koran, c. ii. iv. v. 17. 22. Sale's "Preliminary Discourse." Sec. 6.

§ Lane's "Modern Egyptians," c. iii.

|| Winer's "Biblisches Realw. Art. Bluträcher."

¶ Simonot's "Lettres sur la Corse." p. 314.

human race in the Noachidæ—and the subsequent dispersion of their descendants, according to their families, to all parts of the earth at the Tower of Babel—and we consider the high antiquity and universality of this law to be perfectly confirmatory of our interpretation of the precept. As given "to Noah and his sons," it might have originated partly in the intention of God to express his regard of human life—notwithstanding the late immense destruction of it by the deluge: or it might have been called forth by the "violence" that had "filled the earth" in the anarchical and depraved state of mankind for some ages anterior to the deluge. Many commentators remark that the precept was suited to an infantine state of society, in all respects. It might have been well adapted then, as it is even now, in imperfect states of society, to prevent bloodshed, by committing the revenge of it to the next of kin; but in proportion as mankind became more numerous, and society more humanized and rational, the execution of this ancient law would be liable to many inconveniences—as is still the case where it prevails—calling for some such regulations and adjustment of it as were made by Moses and Mahomet; but that it was merely a *positive* precept, an especial enactment arising out of circumstances, like the law of quarantine, or laws of excise, or particular taxes, and not a *moral* law, or a law founded upon the moral nature of actions and the propriety of relations, and *therefore unalterable*, will be shown subsequently in our observations upon the divine procedure in the case of Cain.

Thirdly. If, indeed, any authority for capital punishment in the case of murder is derivable from this precept, it can only in fairness be taken from the later and improved form of it prescribed by the Levitical law; but, as already observed, such a derivation would, in all fairness, involve the adoption of the whole of that law; and it may be remarked, that the adoption of this particular part of it, with all its adjuncts, would be impracticable in this age and country. This part of the Levitical law was doubtless well adapted to the circumstances of the Jews when they received it, who were then a merely nomadic people—a nation of recently emancipated slaves, to whom a reformatory discipline would have been inconvenient, and who, as many of the enactments of that law clearly show, were not elevated in morals above their late heathen masters, the Egyptians, or their heathen neighbours while in the wilderness, and who were peculiarly intractable; and both then and during some later ages were placed under what Josephus terms a theocracy,* and could instantly consult the supreme lawgiver as to the propriety of inflicting death in any particular instance; and which lawgiver

* Contr. Apion. Book II. c. xvii.

himself was ever supposed to be present at the execution of the sentence. Nor can we forget that the Levitical law was pronounced imperfect, at least in one point, by the Saviour himself—namely, in regard of the provision it made for divorces, of which point, he says, "Moses suffered," or allowed it, "because of the hardness of your hearts;" and it is most worthy of notice that our Lord corrects that enactment by an argument taken from the state of things, "*when God made man*," and "*at the beginning*."* It is also remarkable that no enactment against suicide occurs in the Levitical code, although, as we have already seen, it was probably forbidden in the precept delivered to Noah.

Fourthly. It is, however, sometimes urged that *because the reason of the precept* given to Noah *is general*—namely, "for in the image of God made he man"—therefore the punishment of the murderer with death ought also to be general, since every man that is now murdered was "made in the image of God." Now, we might with perfect satisfaction to ourselves reply to this argument by simply protesting, along with Michaelis, against inferential laws derived from the ancient laws of the Scriptures. We well know the dangers attending such an arbitrary exercise of human judgment on the divine statutes. We give, as an illustration of that danger, the following comment, on the precept in question, by the venerable Bp. Patrick:—"*By parity of reason*, what was ordained against murder was to be executed against other great offences; there being some things which are no less dear to us than life, as virginal chastity and matrimonial fidelity, *&c.*" This "parity of reason" will, of course, seem more or less clear, and more or less extensive to different minds, and, consequently, punishments based upon such an inferential interpretation might extend, especially if various minds were consulted, to an amount, all included within the Bishop's *et cetera*, that would exceed the demands of the most Draconian legislators of modern ages. But, waiving for a moment, both our protest and all that has been already advanced respecting this precept, it may be remarked, in passing on to our more conclusive reply to the inferential use of "the reason for this precept," that some dubiety hangs about the genuine reading, if not even about the genuineness of this part of the precept itself. In the inverted form, "for in the image of God made he man," in which that "reason" appears stated in the English version and in the printed Hebrew text and copies, it looks like a quotation from Gen. i. 27, "So God created man in his own image." But we can hardly conceive of *God* speaking of *himself* in this manner. And it would seem that there is something dubious about this part of the text, since the Septuagint renders the words εν εικονι

* Compare Matt. xix. 3, 7, with Deut. xxiv. 1.

*θεου εποιησα τον ανθρωπον*—because in the image of God I have made man—which, nevertheless, is scarcely suitable to the occasion in which God himself is the speaker. It is still more remarkable that *this* " reason" is not repeated in Leviticus, but that the reason there given for inflicting death upon the murderer is, that the *land* (of the Jews) should not be defiled, and that the land could only be cleansed from " blood by blood."* We will also decline taking any advantage of the dubiety of the Hebrew particle rendered—"*for* in the image"—in this passage, which has sometimes, at least, the sense of *although*. Nor will we resort to any other of the seven different versions which the words in question have received,† nor attempt to convert them into a prediction ; but granting, as Schulz does, the genuineness of the common Hebrew text of this passage, and accepting the English version of it as correct, we urge that the reason here given for the infliction of death on the murderer is simply of a *positive* nature, and, as already observed, it is simply what it seemed good to infinite wisdom and goodness to assign at a particular time, and with a view to a particular effect, and not founded on moral and therefore immutable reasons: and our argument for this view of that "reason" is taken, as heretofore intimated, from the well-known procedure of the Almighty in the case of Cain. For,

Fifthly. " If ever there was a murder," to use the language of modern journalists, " committed by a man in a state of perfect sanity, and with *malice prepense*," it was that committed by the first fratricide. Envy and wrath, arising from the most exceptionable causes, and guiltily unsuppressed, are stated by the Scriptures to have been the causes of the bloody deed. " Cain slew his brother, because his own works were evil and his brother's righteous." " Cain was very wroth, and his countenance fell, because unto Cain and his offering the Lord had not respect; but the Lord had respect unto Abel and his offering."‡ " Perfect deliberation attended the act of unnatural violence," for, according to the reading of the Septuagint, Syriac, Vulgate, and both Targums, a reading pronounced by Dr. Kennicot to be "undoubtedly genuine," although it has entirely slipped out of the present Hebrew text, " Cain said to his brother Abel, let us go forth unto the field." And then, as the English version properly proceeds to read, " it came to pass, when they were in the field, that Cain rose up against Abel his brother and slew him." *But what was the punishment inflicted on Cain?* Instead of being put to death by the

* Numbers, xxxv. 33.

† See Leone Levi's " Law of Nature and Nations." Note.

‡ 1 John iii. 12. Genesis iv. 4, 5, &c.

immediate hand of God, or even by Abel's brother, son, or other next of kin, or by any other human being, "because in the image of God made he man," Cain was simply condemned by the Almighty to disappointment in agriculture, and to be a fugitive and a vagabond in the earth. "The Lord said unto Cain, What hast thou done? The voice of thy brother's blood crieth unto me from the ground. And now thou art cursed from the earth, which hath opened her mouth to receive thy brother's blood from thy hand. When thou tillest the ground, it shall not henceforth yield unto thee her strength—a fugitive and a vagabond shalt thou be in the earth." And when Cain complained of the severity of his sentence, saying, "My punishment is greater than I can bear. Behold thou hast driven me out this day from the face of the earth (land), and from thy face shall I be hid; and I shall be a fugitive and a vagabond in the earth—and it shall come to pass, that every one that findeth me shall slay me. The Lord said unto him, Therefore, *whosoever* slayeth Cain, vengeance shall be taken of him sevenfold. And the Lord set a mark upon Cain"—or, rather, appointed him a sign, that is, some miraculous token—"*lest any* finding him should kill him." Now we maintain, that had the punishment of death, for even a wilful, premeditated, and deliberate murder been founded in *moral* law—that is, in those abstract relations arising from the nature of things, and in the distinctions of moral right and wrong, which, along with all sound authors, we hold to be not the dictates of the mere or sovereign will of the Deity —either the Almighty himself, whose own "everlasting righteousness" consists in his invariable adherence to those moral distinctions, and upon whose inflexible adherence to them all the confidence, all the hopes, and all the fears of his rational creatures are entirely, and will for ever be founded—would either have himself inflicted death upon Cain, or would have required "the blood" of Abel, at the hand of some human being, by requiring him to inflict death upon the fratricide. But since Cain was not put to death by the first of these means, and was actually preserved by a miraculous interposition of God from death by the second of these means, we infer, with entire confidence, that the "reason" of the precept given to Noah, namely, "for in the image of God made he man," is not founded on moral and immutable grounds; that consequently, the precept itself, as well as the reason for it there assigned, are simply of the nature called *positive*, and therefore, and in the absence of any declaration of Scripture to the effect, *not* binding upon the whole human race in all ages. Accordingly, a judicious commentator* is actually driven into the following explanation of the case of

* Pyle.

Cain as compared with the precept to Noah. "I so far forgave Cain the first act of murder, as not to punish him by a violent death for it; *yet, for the future,* life shall go for life."

It cannot be rejoined that there was then no man to kill Cain, for otherwise Cain's dread that any one finding him should slay him, and God's interposition in giving him a sign, *lest* any finding him should slay him, would both have been perfectly futile. It is true that we have no *recorded* account of the existence of more human beings at this period than Adam, Eve Cain, and Abel—neither is the existence of more human beings denied; but it is plainly implied, in the terms of the narrative. Nor is the age of the two brothers respectively recorded; we may safely conceive of them as being each more than a century old. Mr. Scott observes, "Adam and Eve had many more children than are mentioned in the brief narrative, which was principally intended to record a few important particulars, and to trace the history from the beginning to the time of Moses; and if, as is generally thought, Abel was murdered but a short time before the birth of Seth, the human race might have been greatly increased in the space of 130 years."* Stackhouse remarks, "It has been calculated that, according to the Hebrew chronology, there might have been upwards of 420,000 men alone then living, without reckoning women, or even children under seventeen years old; and, if the Septuagint chronology of Dr. Hales be followed, the number of mankind before the death of Abel might have been much greater."† Abel himself might have been the progenitor of a numerous offspring, and Cain also might have been the father of many more children beside Enoch, after whose "name they called the city which he built, after he went out from the face of the Lord and dwelt in the land of Nod." Assuming then that the human race had become numerous, it would seem certain from the dread of Cain that "*every one* finding him should slay him," that the law of blood revenge, as already described, was unknown at that period. Nor does it seem to have been known in regard of the apparently unintentional homicide by Lamech,"‡ who observes, "If Cain shall be avenged sevenfold, truly Lamech seventy-and-sevenfold." Neither is there any trace of this law before the deluge. Since then the infliction of death on Cain for the murder of Abel was prevented by an especial intervention of the Almighty, we are persuaded that the appointment of that punishment to "Noah and his sons" by the hand of the "brother" or next of kin of the murdered man was a mere positive precept;

* Commentary on Genesis.
† History of the Bible. Edit. Bishop Gleig. London. 1817. p. 114.
‡ Genesis iv. 23.

and since the positive precepts of the Levitical law into which that precept was incorporated are not binding upon Christians, so neither is this precept itself, as modified by that law, binding upon Christians. We apply to this precept our Lord's refutation of the Levitical law of divorce, "From the beginning it was not so;" consequently we believe that neither the precept in question, nor any other portion of Scripture, *necessitates* or *compels, proprio vigore,* Christian nations to punish the murderer with death, and that the abolition of this punishment for murder would not be contrary to the inspired and universally obligatory will of God. We regard this precept as partaking in all respects of the same positive character with its associated precept not to eat blood, with the distinction of clean and unclean beasts in the preceding chapter, polygamy, concubinage, and other ante-levitical and positive, and therefore changeable customs, which Moses either incorporated into his legal code as he found them, or regulated by certain restrictions and distinctions, so as to make them useful under the peculiar circumstances of the Jews—but as having no claim upon the universal adoption of Christian nations, because not re-enacted by divine authority upon such nations. Accordingly, we think that to insist upon the adoption of this precept by Christian nations, and especially along with the rejection of other associated precepts, is an act partaking largely of that fondness for "beggarly elements," against which St. Paul set himself with entire and unwearied opposition.

Sixthly. It may be here permitted to the writer to explain his views of the exact ground taken by the *Church* in regard of the question of capital punishment for murder. He craves this permission, because he has often heard it intimated that the Church affords the chief obstacle to the abolition of that punishment for that crime. Now, the Church certainly teaches, in her Thirty-seventh Article, that "the laws of the realm *may* punish Christian men with death for heinous and grievous offences." It is, however, obvious to remark, that the Article does not specify any particular offences, nor does it say *must* punish with, &c.; it speaks only generally and permissively, and it speaks justly here, in its character as "a witness and keeper of Holy Writ;" but in this case it quotes no passage of Scripture; nor is capital punishment for any crime *forbidden* in Scripture. Certain emergencies are easily conceivable in which "the laws of the realm" would be scripturally justified in taking the life of the grievous and heinous offender, and such an emergency would be any offence that was irrepressible by any other means, or not equally repressible; for, as Bishop Burnet observes on this Article, "the lives of men ought not to be too lightly taken except as it appears *necessary for the preservation and safety of the society.*"* It being clear, then, that the Article of the Church does not *necessitate* the punishment of Christian men with death for murder, and it seeming evident to us that neither do the Scriptures unconditionally demand it, we beg to suggest that the *experiment* might be, on all grounds, safely made, whether any or what other mode of preventing murder would at least be equally effectual with the gallows. At all events, we can scarcely imagine that cases of murder would exceed, while such an experiment was being made, in number and atrocity, those which have been perpetrated in Great Britain during the past year, and many previous years, under the full operation of capital punishment.

Nor shall we shrink from the task of proposing an experimental substitute for that punishment for the crime, although such a task is not strictly required by the foregoing investigation. We recommend, then, the confinement of the convicted murderer for life, as a being who is unqualified for the advantages of society. Treat him in all respects as a convict; exact from him hard labour, if he can endure it; but also afford him moral and religious instruction, that, if he be of sound mind, the agency of conscience and the grace of God may effect his genuine repentance unto that "eternal life which no murderer hath abiding in him."† Nor do we believe that the disposal of murderers by this method would increase their crime. From inquiries we have diligently made, we infer that depraved and desperate minds actually dread condemnation to such a mode of spending the rest of their days, more than they dread a violent death. We are happy to find the following concurrence with these latter views in the work of a celebrated writer:—"It is not the *intenseness* of the pain which has the greatest effect on the mind, but its *continuance;* for our sensibility is more easily and more powerfully affected by *weak* but *repeated* impressions, than by a *violent* but *momentary* impulse. The death of a criminal is a terrible but momentary impulse, and therefore a less efficacious method of deterring others than the *continued example* of a man deprived of his liberty, and condemned as a beast of burden to repair by his labours the injuries he has done to society. 'If *I* commit such a crime, says the spectator to himself, I shall be reduced to that miserable condition for the rest of my life!' A much more powerful preventive than the fear of death, which men always behold in distant obscurity. In the contemplation of *continued* suffering, *terror* is the only, or, at least, the permanent sensation.‡"

* Exposition of the Thirty-nine Articles.
† 1 John iii. 15.
‡ Marquis de Beccaria's "Essay on Crimes and Punishments." Edit. 2.

Although we are fully aware that the readers of this journal are not influenced merely by names, yet it may be acceptable to them to find that some divines of the highest eminence, and belonging to various communions, have coincided with our interpretation of the precept given "to Noah and his sons." Nor are we surprised to find some commentators, especially English, interpreting that precept as a universal and perpetual command for putting the murderer to death. Our very few English commentators on the whole Bible, probably viewed this precept under the bias of national prepossessions. Nor are commentators upon the whole Bible, or on any very considerable portion of it, the best referees for the sense of individual portions of Scripture. The vastness of their undertaking does not admit of their scrutinizing particular passages with sufficient pains. Such commentators are generally mere compilers: they follow the beaten track of interpretation, as even Whitby himself ultimately confessed he had done* with regard to his commentary on the New Testament. The opinion of one unbiassed, learned, and investigating divine is worth more than a whole host of adopted, retailed, and conventional interpretations. We proceed to adduce the opinions of the superior class of theologians, in addition to those quoted in the preceding paper. Arnheim gives his opinion that "the precept establishes the *lex talionis:* if one stranger האדם slay another, the kinsmen of the murdered man are the avengers of blood; but if he be slain by איש אחיו *one of his own kindred*, the other kinsmen must not spare the murderer; but should they do so, then Divine Providence will require the blood, *i.e.*, avenge it." Schulz remarks upon the precept—"God is here speaking to men having no magistrates. Hence, he grants to the whole society of men, and to every individual man, the right and power of punishing any homicide, whatever, with death, and at the same time inculcates the use and exercise of this right and given power. The society of men living in a natural state requires it, and it is exercised by all nations where they live in a state of nature without magistrates. But this law, given and inculcated upon men living in a state of society, which readily runs into abuse, *does not reach beyond; neither, therefore, is a positive, universal law to be deduced from this passage.*"† Michaelis remarks upon the application of this precept to "the infancy of society," and "its antiquity long before the time of Moses, who adopted the wise plan of appointing the privilege of asylums, as did most other legislators; thereby taking away, in a great degree, the power to punish the murderer from the Goel, and preventing, what must have often happened, the shedding of innocent blood."‡ Houbi-

* Whitby's "Last Thoughts." † Scholia in Vet. Test. ‡ Recht Moses.

gant applies the precept to "the law of retaliation, and not to punishment inflicted by the magistrates." Dr. Geddes adopts the same view of it. To these authorities we may add those of Winer and Gesenius. After all, the question must be left to the judgment of the reader, formed upon a consideration of the materials now submitted to it, and directed by the enlightened benevolence, which it is the privilege of a sound understanding to enjoy and exercise under the present advanced state of Christian knowledge, science, reason, and civilization.

---

